# Long-Term Water Quality Patterns in an Estuarine Reservoir and the Functional Changes in Relations of Trophic State Variables Depending on the Construction of Serial Weirs in Upstream Reaches

**DOI:** 10.3390/ijerph182312568

**Published:** 2021-11-29

**Authors:** Namsrai Jargal, Ho-Seong Lee, Kwang-Guk An

**Affiliations:** Environmental Ecology Laboratory, Department of Bioscience and Biotechnology, Chungnam National University, Daejeon 34134, Korea; jargal.namsrai.sci@gmail.com (N.J.); hosung5419@naver.com (H.-S.L.)

**Keywords:** Yeongsan Reservoir, cultural eutrophication, rainfall intensity, Seungchon and Chucksan weirs, long-term time-series, spatial and seasonal patterns

## Abstract

Water quality degradation is one of the major problems with artificial lakes in estuaries. Long-term spatiotemporal patterns of water quality in a South Korean estuarine reservoir were analyzed using seasonal datasets from 2002 to 2020, and some functional changes in relations of trophic state variables due to the construction of serial weirs in the upper river were also investigated. A total of 19 water quality parameters were used for the study, including indicators of organic matter, nutrients, suspended solids, water clarity, and fecal pollution. In addition, chlorophyll-a (CHL-a) was used to assess algal biomass. An empirical regression model, trophic state index deviation (TSID), and principal component analysis (PCA) were applied. Longitudinal fluctuations in nutrients, organic matter, sestonic CHL-a, and suspended solids were found along the axis of the riverine (Rz), transition (Tz), and lacustrine zones (Lz). The degradation of water quality was seasonally caused by resuspension of sediments, monsoon input due to rainfall inflow, and intensity of Asian monsoon, and was also related to intensive anthropic activities within the catchment. The empirical model and PCA showed that light availability was directly controlled by non-algal turbidity, which was a more important regulator of CHL-a than total nitrogen (TN) and total phosphorus (TP). The TSID supported our hypothesis on the non-algal turbidity. We also found that the construction of serial upper weirs influenced nutrient regime, TSS, CHL-a level, and trophic state in the estuarine reservoir, resulting in lower TP and TN but high CHL-a and high TN/TP ratios. The proportions of both dissolved color clay particles and blue-green algae in the TSID additionally increased. Overall, the long-term patterns of nutrients, suspended solids, and algal biomass changed due to seasonal runoff, turnover time, and reservoir zones along with anthropic impacts of the upper weir constructions, resulting in changes in trophic state variables and their mutual relations in the estuarine reservoir.

## 1. Introduction

Water quality plays an important role in providing sustainability for human use and ecological balance within reservoirs [1]. This is associated with a variety of water quality parameters that determine the physical, chemical, and biological components, as well as their relationships. Accordingly, long-term water quality monitoring based on appropriate parameters provides important information on the status of reservoirs undergoing anthropogenic and natural impacts, as well as contributing efficient plans of water resources and conservation of aquatic life [2]. 

An estuary is a transition zone between coastal water and river mouth [3]. Water quality degradation in the estuary is one of the most vulnerable environmental problems due to the high release of agricultural, domestic, and industrial effluents [4,5,6]. Moreover, some dams are constructed in this transition region to provide a number of socio-economic benefits such as flood control, irrigation water, hydroelectric generation, storm surge mitigation, and permanent freshwater source [7]. Although they meet the needs of the people, there are water quality problems in estuarine systems related to the dams. For instance, there is an accumulation of pollutants, oxygen depletion due to thermal and saline stratification and eutrophication [7,8]. High sedimentation rate due to dams also reduces turbidity in the water column of lentic systems. As a result, severe algal blooms occur in the systems, especially when sufficient nutrients are present and there is a long residence time [9].

Spatial and seasonal pattern analyses are important for water quality management in reservoirs. This is because they exhibit seasonally and spatially varying characteristics due to internal and external factors [10,11,12]. Their spatial variations are determined by longitudinal gradients in the physical, chemical, and biological factors. It can be generally patterned as three longitudinal zones, which are the riverine zone (Lz), transition zone (Tz), and lacustrine zone (Rz) [12]. Meanwhile, their physicochemical properties vary seasonally depending on temperature and precipitation [4,9]. The intensity of rainfall during the Asian monsoon (July–August) has a great influence on water quality in Korean reservoirs [13,14]. As a result of monsoonal rainfall, high inflow and outflow rates, low residence time of water, and excessive levels of pollutants (organic matter and nutrients, etc.,) are observed, accompanied by anthropogenic runoff [11,14,15]. Due to nutrient overload from intensive agricultural land use, the Korean reservoirs are particularly at risk of massive algal blooms [16]. Therefore, cultural eutrophication is still major problem for aquatic systems in the Korean Peninsula [16,17], especially estuaries. The resulting impacts give rise to water problems such as harmful algal blooms, impairment of recreational opportunities, oxygen depletion, and loss of aquatic organisms [18,19]. 

Chlorophyll-a (CHL-a) is the main indicator of algal biomass in aquatic environments and is one of the most important parameters for assessing eutrophication in reservoirs, as it is closely related to various environmental factors [20]. Specific studies on the algal biomass-nutrients relations have been conducted for nutrient management strategies to mitigate cultural eutrophication in reservoirs or lakes worldwide [13,21,22]. Most studies based on ambient concentrations suggested that CHL-a was more strongly associated with total phosphorus (TP) than with total nitrogen (TN). This supports the view that phosphorus is an important regulator of algal growth in water bodies [22,23,24]. However, the linear relationships between TP and CHL-a in systems can be differed by latitudinal variation, spatial heterogeneities of physicochemical factors, and land-use pattern [16,25,26,27]. In addition, the algal growth in estuarine systems can be light-limited due to high inorganic turbidity [7]. The trophic state index is commonly applied to assess the environmental health and trophic condition of lentic systems [28,29]. The most commonly used method is Carlson’s trophic state index (TSI), which is based on TSI (TP), TSI(CHL-a), and TSI(SD) [30]. Additionally, the interrelationships among the variables of TSI in line with deviations of TSI(CHL-a)-TSI(TP) and TSI(CHL-a)-TSI(SD) can be used to investigate certain situations and limiting factors in reservoirs or lakes [28,31]. In addition, dissolved oxygen (DO) is an important variable for assessing the water quality of estuarine systems. Its spatial-seasonal variances depend on multiple physical and biochemical processes as well as human-induced problems [32]. Hypoxia due to a reduction in the DO impacts severely aquatic health and integrities, for example, toxic algal blooms and loss of biotic integrity [32,33]. Its concentrations below 5 mg/L can negatively affect the functioning and survival of biotic communities and below 2 mg/L may lead to the death of most fish [9]. These conditions are mainly caused by the issues in line with anthropogenic effluents into the aquatic ecosystems [4].

The Yeongsan Reservoir is located at the mouth of the Yeongsan River in western South Korea. Its dam was commissioned in 1981 and is used for flood control, reclaiming of tidal mudflats, and agricultural and industrial water use. However, the estuarine reservoir is still facing continuous and seasonal water quality problems such as oxygen depletion [15,34], high rate of sedimentation, increased anthropogenic discharges (organic matters and nutrients), and eutrophication [15,35,36,37]. Accordingly, a previous study reported fish diversity loss in the system [7]. Therefore, further scientific assessments of the spatial-seasonal and long-term patterns of water quality are needed for appropriate management. In addition, two serial weirs were constructed at the upstream reaches of the reservoir in 2011 (Seungchon and Chucksan) as part of a restoration project for four major rivers [38]. These serial weirs could affect physicochemical and trophic conditions in the estuarine reservoir according to the concept of serial discontinuity [39]. However, there is a lack of information on how the construction of upstream serial weirs affects the physicochemical patterns and trophic parameters relations in estuarine reservoirs.

In connection with the loss of reservoir water quality and the construction of serial upper weirs, we analyzed here the 19-year times series of seasonal water quality datasets in three longitudinal zones of the estuarine reservoir. A total of 19 water quality variables were used for this study. The first objective was to describe spatial seasonal water quality variations in the reservoir, as well as to identify the main drivers of the changes. Moreover, we determined potential key factors influencing algal biomass in the reservoir. The second objective was to investigate the impact of the construction of serial headwater weirs on the trophic state variables and their functional relations in the estuarine reservoir. 

## 2. Materials and Methods

### 2.1. Study Area and Sites

**Figure 1:**
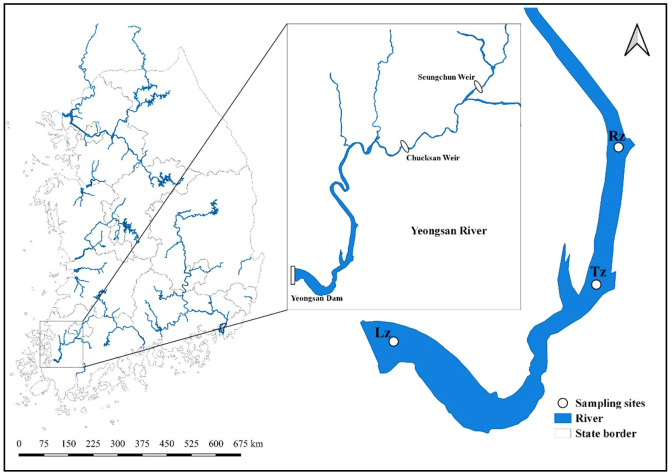
Water quality sampling sites in the Yeongsan Reservoir as considering the longitudinal zones (Rz—riverine zone, Tz—transition zone, and Lz—lacustrine zone).

Our study reservoir is located at the mouth of the Yeongsan River in southwest Korea (Figure 1). In terms of geographical location, the estuarine system is located in a temperate climate zone of the Northern Hemisphere. The winter (December–February) is influenced by cold and dry northwesterly winds under the Siberian high pressure. Drought in spring (March–May) is associated with northeasterly winds caused by anticyclones. The influence of the North Pacific high-pressure system contributes to hot and humid weather during summer (July–August) with intense rainy [14]. 

The reservoir has a surface area of 34.6 km^2^ and an average depth of 10.1 m. Its dam was commissioned in 1981 for flood and tidal mitigations, irrigation water, and industrial water supply. Its catchment is predominantly used for agricultural, industrial, and urban activities. Consequently, numerous point and non-point discharges load into the reservoir from the catchment, leading to water quality problems such as oxygen depletion and cultural eutrophication [7,15,34,35]. In 2011, two weirs, Seungchon and Chucksan, were built at the upstream reaches of the reservoir as part of a restoration project of four major rivers. Considering the depth-related longitudinal gradients of water variables in reservoir systems [11,12], we selected three monitoring sites in the Yeongsan Reservoir (Figure 1), which are the lacustrine zone (Lz), transition zone (Tz), and riverine zone (Rz). The Lz shows similar structural patterns to natural lakes caused by the deepest and broadest area located near dams. However, the Rz is a narrow and fluvial area at an upper reach of reservoirs. Thus, its condition resembles more parent rivers. The Tz is between the lacustrine and riverine zones.

### 2.2. Long-Term Time Series Data 

Long-term seasonal data on water quality parameters at each site, from 2002 to 2020, were obtained from the National Institute of Environmental Research (NIER). A total of 19 water quality variables were used for further water quality analyzes. The variables included water temperature (WT), electric conductivity (EC), total suspended solids (TSS) Secchi depth (SD), non-algal turbidity (Kna), the potential of hydrogen (pH), dissolved oxygen (DO), biological oxygen demand (BOD), chemical oxygen demand (COD), total nitrogen (TN), total dissolved nitrogen (TDN), ammonium-nitrogen (NH_4_-N), nitrate-nitrogen (NO_3_-N), total phosphorus (TP), total dissolved phosphorus (TDP), orthophosphate (PO_4_-P), TN:TP ratio, fecal coliform bacteria (FCB), and chlorophyll-a (CHL-a). 

The WT, EC, DO, and pH were measured in situ using a portable multiparameter analyzer (YSI Sonde Model 6600). The SD is a measure of water transparency (water transparency). It was measured using a 20 cm disk with alternating black and white quadrants. Nutrients were analyzed using the standard methods of the Ministry of Environment, South Korea [40]. The TN was measured using a UV spectrophotometric method based on potassium sulfate digestion. Ammonium nitrogen (NH_4_–N) was analyzed at 630 nm using the phenate method after the water sample was filtered through GF/C filters, and nitrate-nitrogen (NO_3_–N) was measured using the ion chromatography method after the water sample was filtered. The TP was quantified with unfiltered water digested by the ascorbic acid method after persulfate oxidation, and PO_4_-P was determined without digestion with filtered water by ascorbic acid methods. The TDP was determined by the ascorbic acid method with the digestion of filtered water. The BOD, COD, TSS, FCB, and CHL-a were measured according to the standard methods of APHA [41]. TSS was determined after drying at 105 °C for one hour. CHL-a concentration was determined after extraction in ethanol using a spectrophotometer. The FCB was counted using the fecal coliform test (EC medium) [41].

### 2.3. Trophic State Index Deviation and Non-Algal Turbidity

Trophic state index deviation (TSID) is a graphical method for identifying the relationships between trophic state variables and limiting factors in lentic systems and for determining specific conditions [28,31]. It is based on the deviations of TSI(CHL-a)-TSI(TP) and TSI(CHL-a)-TSI(SD) [31]. To perform TSID, we calculated Carlson’s trophic state index for TP, SD, and CHL-a using the following equations [30].

TSI (SD) = 60 − 14.41 Ln (SD)(1)

TSI (CHL-a) = 30.6 − 9.81 Ln (CHL-a)(2)

TSI (TP) = 14.42 Ln (TP) − 4.15(3)

Water clarity, as measured SD, can be affected by algal biomass (CHL-a) and a non-algal component in water bodies. Non-algal components include materials such as suspended inorganic matter (clays and silts) and water color. The CHL-a is a poor predictor of SD in some lentic systems due to turbidity fluctuations caused by non-algal matter [42]. In meantime, turbidity can limit algal growth due to reduced light availability. According to the main reference [42], non-algal turbidity (Kna) was determined from the seasonal averages of SD and CHL-a using the following equations.

K_na_ = 1/SD − 0.025 × CHL-a (4)

### 2.4. Statistical Approaches

An ANOVA test was applied to evaluate the seasonal variation of water quality parameters in the longitudinal zones, as well as the long-term changes due to the construction of serial upper weirs. The empirical regression model was used to determine the main factor influencing algal biomass and water clarity (or transparency). The graphical plots and regression model were performed using SigmaPlot V.14.5 (Systat Software Inc., San Jose, CA, USA). Principal component analysis (PCA) is frequently and successfully applied in the field of water quality analysis to uncover relationships between water quality parameters and spatial-seasonal pollutant sources’ determination [43]. Here, PCA was performed using the functions “princomp()” and “ggbiplot” in R software (R 4.0.3 version). It was based on the monthly average values of the respective parameters. The criteria of eigenvalue [44] helped in determining the principal components (PC) that interpret most of the variations in water quality. The loading values of water quality variables in the significant components were classified as large (>0.70), medium (0.70–0.50), and small (0.50–0.30) [45]. 

## 3. Results and Discussion

### 3.1. Seasonal Heterogeneities of Water Quality Parameters on the Longitudinal Zones in the Reservoir 

Reservoirs exhibit a pronounced gradient of physicochemical properties from lotic to lentic conditions, depending on a variety of hydrologic patterns such as flow regime, water level, depth, and surface area [46,47]. Meanwhile, the water quality of the systems changes in a seasonal-specific manner depending on regional climatic factors such as temperature and precipitation [4,10,46].

All physical parameters showed significant variations (*p* < 0.001) along the longitudinal zones except WT (Supplementary Tables S1 and S3). TSS and K_na_ decreased from the Rz to the Tz and to the Lz, while EC and SD increased significantly. The longitudinal decrease in TSS and Kna was inferred from a high rate of suspended sediment sinking to the bottom near the dam [11,46,47], resulting in increased water clarity in the Lz. The increase in EC was generally related to the influence of coastal water salinity on the estuarine reservoir. This is because the EC is sensitive to variations in dissolved solids, mainly mineral salts [10]. However, in non-estuarine reservoirs of Korea, an increasing trend of EC from Lz to Tz and to Rz was generally observed, as EC is more correlated with anthropogenic pollutants [45,48,49,50].

**Figure 2:**
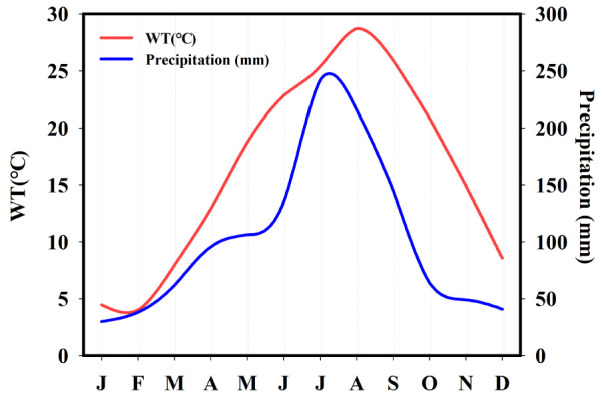
Monthly averages (January (J) to December (D)) of precipitation and water temperature (WT) of the reservoir.

**Figure 3:**
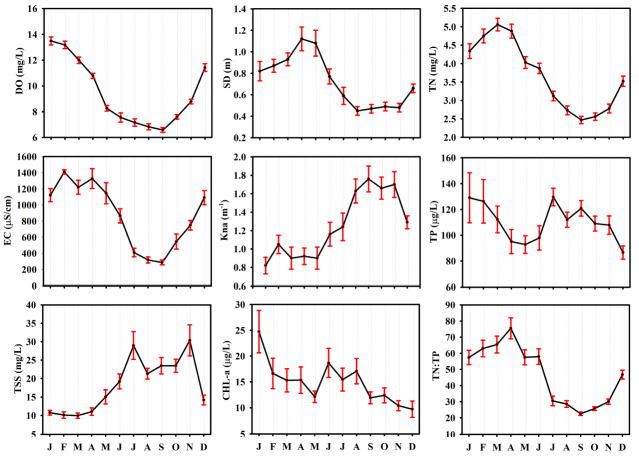
Monthly dynamics (January (J) to December (D)) of water quality parameters were presented by their averages and standard error (SE) during 2002–2020 (DO—dissolved oxygen, EC—electric conductivity, TSS—total suspended solids, SD—secchi depth, Kna—non-algal turbidity, CHL-a—chlorophyll a, TN—total nitrogen, TP—total phosphorus).

Seasonally, all physical parameters also showed significant variation in each longitudinal zone (*p* < 0.001, Supplementary Tables S2 and S3). The EC in each zone was low in summer and autumn, but high in winter and spring. The lowest average value was 272 μS/cm in summer in the riverine zone (Supplementary Table S2). These results indicated that the seasonal sharp decrease in EC (dissolved solids and salts) was mainly influenced by the high input of freshwater (through ionic dilution) into the system due to monsoon rainfall [4,37,51,52]. The WT, TSS, and Kna were high in each longitudinal zone in summer and autumn, while they were low in winter and spring (*p* < 0.001). Meanwhile, the monthly dynamics of these parameters confirmed their seasonal pattern. The monthly increase in TSS and Kna was positively related to the precipitation and WT (Figure 2 and Figure 3). The two different factors, namely the sediment resuspension and the anthropogenic runoff, could lead to seasonal increases in TSS and Kna. This is because the estuarine reservoir is facing problems of severe sedimentation and anthropogenic pollutants [7,15,35,36,37,38]. Warm surface water begins to cool and vertical mixing (turnover) occurs in temperate systems, resulting in resuspension of pollutants from sedimentation. As a result, the inorganic suspended solids could increase in temperate estuarine regions [15,53]. On the other hand, intense rainfall occurs in the reservoir region during the Asian monsoon (from July to early September) [37,38], accounting for 54% of the annual rainfall of 1357.7 (Figure 2). Accordingly, anthropogenic pollutant runoffs to the reservoir increase [7,15,37], which could lead to high levels of TSS and Kna in summer and fall. As for the seasonal water clarity, SD was lower in summer and fall compared to in winter and spring in each zone, with a significant statistical difference (*p* < 0.001, Supplementary Tables S2 and S3). Moreover, the monthly dynamics of SD showed that the water clarity in the reservoir was observably influenced by the TSS and Kna (Figure 3), especially in summer and fall. 

Organic matter indicators (BOD and COD) showed significant variation for longitudinal zones (*p* < 0.001), along with their increase from Lz to Tz and to Rz (Supplementary Tables S1 and S3). The BOD showed a significant seasonal difference only in the Lz, as the concentration was lower in autumn than in the other season (H = 22.49, *p* < 0.001, Supplementary Tables S2 and S3). The COD showed significant seasonal differences for each longitudinal zone. In the Rz, the concentration of COD was significantly higher in summer (7.38 mg/L) than in the other seasons (H = 53.02, *p* < 0.001). In the Tz and Lz, the COD levels were significantly higher in summer than in autumn and winter. For the fecal pollution indicator, FCB did not show any statistical difference along longitudinal zones. However, we found a significant seasonal variation for FCB on the Tz and Rz, and their number increased mainly in summer and fall (Supplementary Tables S2 and S3). The seasonal COD and FCB patterns showed that sewage and industrial effluents into the reservoir increased in association with the heavy rainfall [37,54,55,56]. 

The DO was higher in the Lz than in the Tz and Rz (Supplementary Table S1), and there was a statistically significant difference (H = 17.57, *p* < 0.001, Supplementary Table S3). In general, the increase correlated with the significant decreases in organic matter, nutrients, and algal biomass along the axes of Rz, Tz, and the Lz. The seasonal pattern showed significant variation in each longitudinal zone. The concentration was significantly lower in summer and winter than in the other two seasons (Supplementary Tables S2 and S3). The lowest average of DO (6.08 mg/L) was recorded in the Tz during summer. Furthermore, the monthly DO in the reservoir decreased noticeably from January (13.49 mg/L) to September (6.58 mg/L, Figure 3). The monthly and seasonal pattern showed the influence of anthropogenic and natural processes on the DO in accordance with the dynamics of WT and precipitation (Figure 2), such as flow advection, pollutant loads, and biological activities [7,15,32,34].

Phosphorus and nitrogen are ecologically important nutrients for the water system and often limit algal growth in reservoirs and lakes. However, excessive nutrient loadings from the watersheds are considered eutrophication [18,32,57]. In our study, TP decreased significantly along the longitudinal zones (from Rz to Tz, and to Lz), but TDP and PO_4_-P did not show statistically significant differences (Supplementary Table S3). The nitrogen contents (TN, TDN, and NH_4_-N) showed the significant variation through the studied zones (H = 11.49, 10.18, respectively, and 19.02, *p* < 0.001,) associated with these lower concentrations in the Lz than in the Tz and Rz (Supplementary Tables S1 and S3). The ambient ratio TN/TP elucidated the large statistical variations among the zones, together with its decrease from Lz to Tz, and to Rz. 

As for the seasonal pattern of nutrients, nitrogen contents (TN, TDN, NH_4_-N, and NO_3_-N) showed a large statistical difference among longitudinal zone (*p* < 0.001, Supplementary Tables S1 and S3). Their concentrations typically decreased from spring to summer, summer to fall, and increased from fall to winter (Supplementary Table S2). In particular, their concentrations were relatively lower in summer and fall than in spring and winter. The monthly TN in the reservoir reflected the seasonal pattern. The monthly decrease in TN was connected with the increase in monthly precipitation (Figure 2 and Figure 3). The outcomes support the influence of ionic dilution on the nitrogen contents in connection with large amounts of rainfall [7,48,49,50,58]. The seasonal patterns of phosphorus contents (TP, TDP, PO_4_-P) in each zone showed no statistically significant differences. However, the monthly variations of TP in the reservoir showed that the concentration increased in the months with heavy rainfall (July–September) and was also high in winter (Figure 2 and Figure 3). The seasonal and spatial mean values of TP showed a eutrophic condition in the Lz and a hypertrophic condition in the Tz and Rz (Supplementary Table S1) according to the criteria of Carlson [28]. As for the ratio TN:TP, its seasonal value changed significantly in each longitudinal zone (*p* < 0.001). The ratio was significantly lower in summer and fall than in winter and spring. Typically, the low TN:TP ratio is observed in lentic systems with intense anthropogenic activities and a high trophic status [13,26]. The low ratio (>29) may also indicate the presence of cyanobacteria (blue-green algae) occurrence in water bodies [59]. Strictly low monthly average values in the reservoir occurred during the months of August to October compared to the other months (Figure 3). The respective averages were 28.57, 22.52, and 25.77. As indicated by the result, the occurrence of blue-green algal in the estuarine system probably showed an increasing trend during summer and fall.

We used chlorophyll-a concentration to assess the algal biomass in the system. This is because the resulting high concentrations would lead to large productivity values and high algal biomass in water bodies [22]. CHL-a in the estuarine reservoir increased significantly from Lz to Tz, and to Rz (H = 58.06, *p* < 0.001, Supplementary Tables S1 and S3). Their respective mean values were 9.56 μg/L, 12.37 μg/L, 21.87 μg/L, indicating a meso and eutrophic condition of the reservoir for CHL-a [28]. However, its concentration did not show significant seasonal differences in each longitudinal zone (Supplementary Tables S2 and S3). As for the monthly variations in the reservoir (Figure 3), CHL-a increased in the summer months related to the high WT, but the highest average value (24.72 μg/L) occurred in January. The highest value might be related to with low non-algal turbidity and high TP in winter (Figure 3), and the long residence time [7,15].

### 3.2. Influences of Nutrients, Non-Algal Turbidity, and Total Suspended Solids on the Reservoir Water Clarity and Algal Biomass 

**Figure 4:**
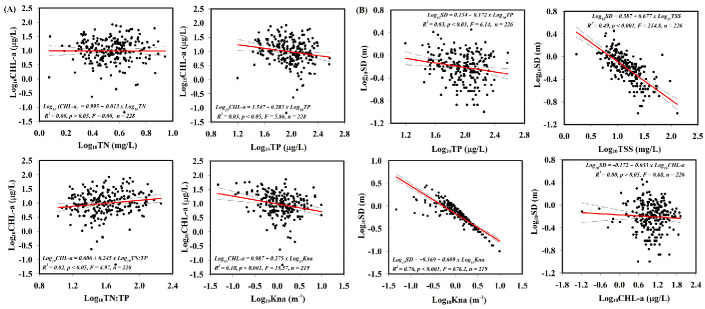
Empirical models of nutrients (TN and TP) and non-algal turbidity (Kna) on the algal biomass (CHL-a) (**A**), and showing the relationship between water clarity and nutrients, Kna, and CHL-a (**B**).

We applied three statistical methods for the investigations in this section. The first approach was the empirical regression model based on the long-term seasonal average values of the respective parameters (TN, TP, TN:TP ratio, Kna, CHL-a, and SD) in the longitudinal zones. Moreover, the average values of these parameters were converted into log-transformed values (Log_10_) to obtain a normal distribution. Previous empirical studies on Korean reservoirs suggested that TP was the best predictor of CHL-a rather than other factors under meso-eutrophic conditions [48,57]. However, our empirical model showed neither nutrients (TN and TP) had a significant correlation with the algal biomass in the estuarine reservoir, as was the case with the long-term time series (Figure 4).

This is likely due to the hypertrophic nutrient condition (especially TP) and high inorganic turbidity. Meanwhile, Kna showed a significant negative effect on algal biomass (*p* < 0.001), while it explained only 10% of the variation in algal biomass variation. For water clarity of the reservoir, SD showed a strong negative relationship with Kna (R^2^ = 0.76, *p* < 0.001) and TSS (R^2^ = 0.44, *p* < 0.004). However, SD did not show any significant correlation with TP and CHL-a (Figure 4).

Secondly, the multivariate statistical approach was used to obtain more interpretations about the influence of the selected factors on water clarity and algal biomass. The parameters included WT, EC, TSS, Kna, TN (its allied components), TP (its allied components), TN:TP, FCB, and the precipitation (PREC) according to their seasonal significant variations and significance. The first four principal components (PC 1, PC 2, PC 3, and PC 4) according to the eigenvalue criteria [44] were used to interpret the relationships among the parameters (Table 1). The selected components explained 56.2%, 17.6%, 13.1%, and 6.73% of the total variance, respectively. The PC1 contained the large positive loadings of EC, SD, nitrogen contents, and TN:TP (Table 1). In contrast, it had large negative loadings of WT, TSS, Kna, and the FCB as well as small negative loadings of particulate phosphorus (PP), PP:TP, and the PREC. As a result, the component showed predominantly seasonal variations in the selected parameters (Table 1 and Supplementary Figure S1). In addition, it suggests that the large seasonal increase in TSS, Kna, and FCB strongly regulated water clarity as well as algal biomass. The PREC regulated moderately the seasonal variation of parameters with large and small loadings in this component, in agreement with increasing processes such as ionic dilution, anthropogenic effluents, and turbidity [7,15,38,50].

The second component contained two large positive loadings of TDP and PO_4_-P as well as a small positive loading of TP. However, the PP, PP:TP, and EC showed small negative loadings. In meantime, there were small positive contributions of the CHL-a and PREC. Therefore, this component mainly showed that the increased inputs of dissolved phosphorus into the estuarine reservoir accompanied by anthropic runoffs from human activities within the catchment due to the high precipitation [7,15,38]. The third component mostly showed when the WT and PREC were the small negative loading, the proportion of PP in the TP largely loaded with the medium positive loading of the algal biomass. As for the first component, the PP in the reservoir was closely related to external inputs caused by monsoonal rainfall, but this component showed that the parameter (PP) was closely related to the algal biomass during winter in the estuarine system. The fourth component contained medium positive loadings of WT and PREC but also being a small positive loading of the algal biomass.

The outcomes of two different analyses showed that the water clarity in the estuarine system was regulated more by TSS and Kna rather than by algal biomass and nutrients. The increase in regulatory parameters was closely related to the external inputs into the reservoir in associated monsoonal rainfall events. Algal biomass tended to respond negatively to increases in TSS and Kna, supporting light limitation in the system [60]. However, it showed a significant positive interaction with loadings of phosphorus in the second and third components. As indicated by these results and their monthly dynamics (TP and CHL-a), phosphorus was probably a more important parameter for the algal growth in the estuarine system during winter [15], resulting in high algal biomass.

**Figure 5:**
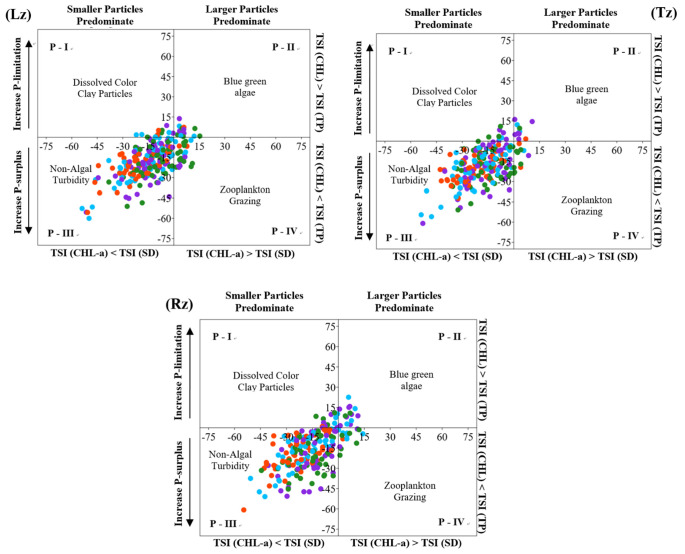
Spatial seasonal pattern of trophic state index deviation (TSID) based on total phosphorus (TP), chlorophyll-a (CHL-a), and secchi depth (SD) on each longitudinal zone (Rz—riverine, Tz—transition, and Lz—lacustrine; colors’ meaning: green—spring, blue—summer, orange—fall, purple—winter).

The third approach was TSID based on the relationship between trophic state parameters [31]. It additionally evidenced that the non-algal turbidity (or inorganic turbidity) dominated in the estuarine reservoir in terms of both spatial and seasonal patterns (Figure 5). The graphical result showed that small proportions of dissolved color particles (P-I), blue-green algae (P-II), and zooplankton grazing (P-IV) occurred in the longitudinal zones. In addition, the proportion of blue-green algae (P-II) in the TSID tended to increase in winter and summer, as well as from the Lz to the Tz and to the Rz.

### 3.3. Impact of Serial Weir Constructions on the Nutrient Regimes, CHL-a, and TSID within the Estuarine Reservoir, along with Monsoonal Rainfall Intensity

The ecological impact of hydrological changes in river ecosystems is importantly connected with water quality issues [61]. Hydrological changes caused by dams and weirs, such as the water level, water velocity, and water residence time, alter physical, chemical, and biological properties of both upstream and downstream [62,63,64]. Our study showed that the construction of upstream serial weirs (Seungchon and Chuksan, which commissioned in 2011) significantly affected the nutrient regimes, algal biomass, and their mutual relationships (ratios of TN:TP and CHL-a:TP) in the estuarine reservoir, as well as the proportion of TSID. 

**Figure 6:**
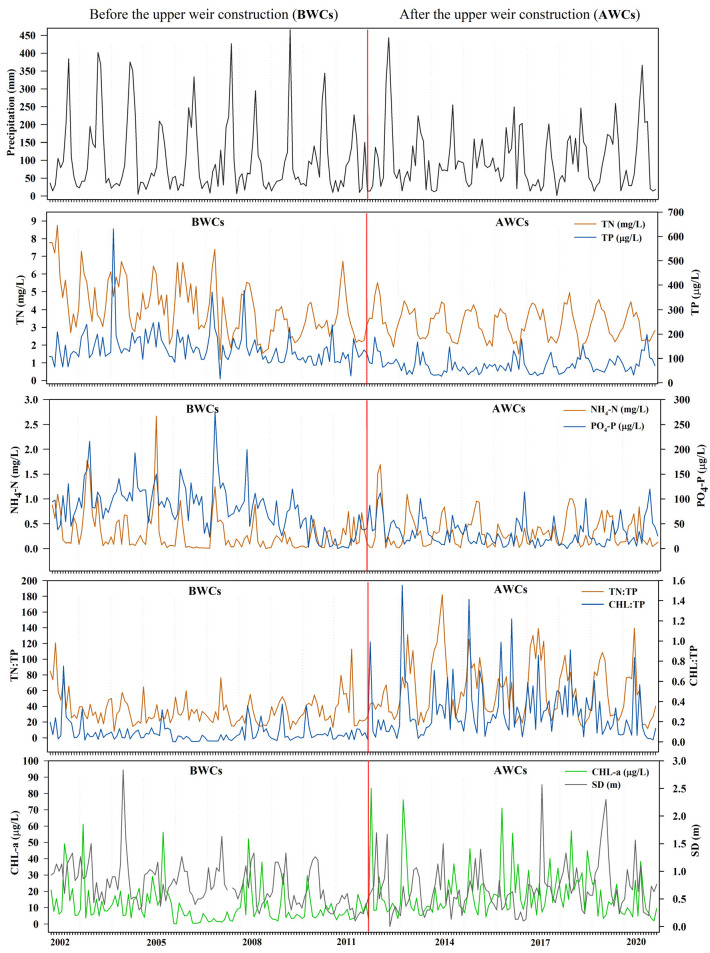
Long-term seasonal variations of the nutrients (TN—total nitrogen, TP—total phosphorus, NH_4_-N—ammonium nitrogen, and PO_4_-P—orthophosphate), algal biomass (CHL-a), secchi depth (SD), and CHL:TP ratio and TN:TP ratio in the reservoir.

The present results showed that most nutrients compounds decreased after the construction of upper weirs (Table 2 and Figure 6). In particular, the average values of TP, TDP, and PO_4_-P in the reservoir were reduced three or four-fold (*p* < 0.01, Table 2). Accordingly, the trophic state for TP has reduced from hypereutrophic to eutrophic [28]. However, the reduced condition was still sufficient to enhance algal growth as in lentic systems. In contrast, CHL-a, ratios of CHL-a:TP and TN:TP significantly increased after the construction of the upper weir (*p* < 0.01, Table 2 and Figure 6). 

Moreover, Figure 6 clearly showed that the pattern and amount of rainfall during 2013–2018 were completely different from 2013. This variance was mainly related to the rainfall intensity during the monsoon season when the rainfall was at least 150 mm lesser. Together with anthropogenic influences of the upper weirs, this could lead to low nutrient levels but high algal biomass in the years 2013–2018. The intensity of monsoonal rainfall primarily controls the annual amount of organic matter and nutrients in the reservoirs of South Korea due to the anthropogenic runoff [11,15,16]. The increase in algal biomass was probably due to the following changes in the estuarine reservoir caused by the above influences; (1) prolongation of water residence time, (2) increase in light availability due to reduction in inorganic turbidity, and (3) sufficient availability of nutrients for algal growth even though nutrients have decreased [62,65].

**Figure 7:**
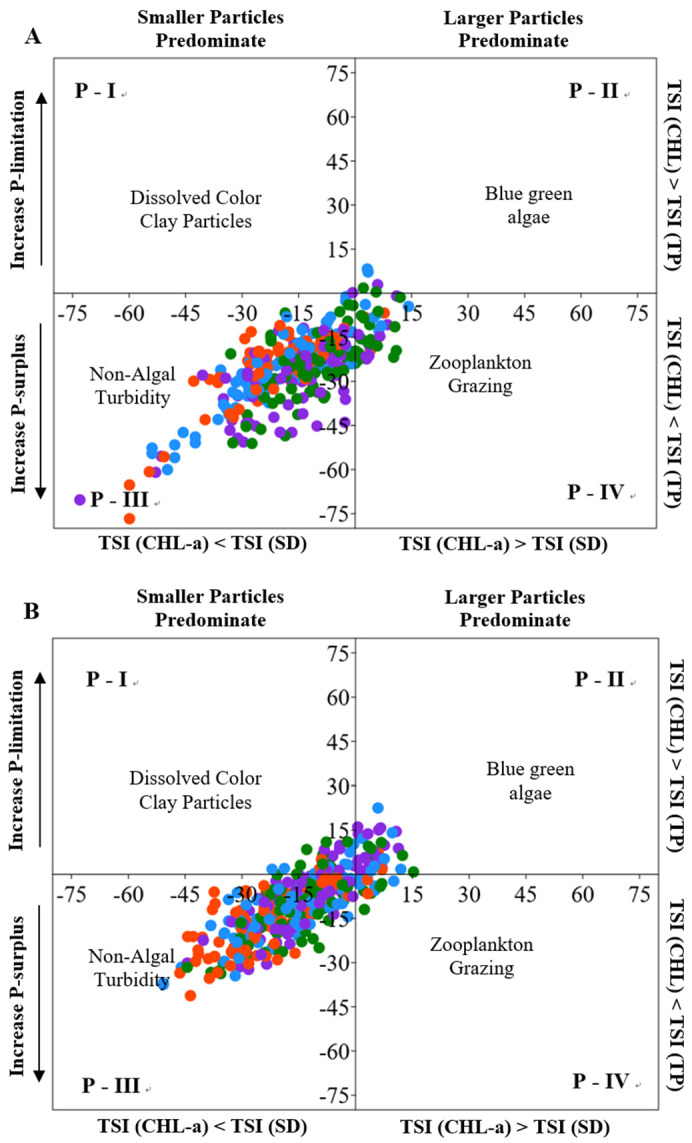
The difference in trophic state index deviation (TSID), based on total phosphorus (TP), chlorophyll-a (CHL-a) and secchi depth (SD), before (**A**) and after (**B**) the upper weir constructions (color meaning: green—spring, blue—summer, orange—fall, purple—winter).

We also examined the variation in the proportion of TSID associated with the constructions of serial weirs (Figure 7A,B). Regarding TSID, the study reservoir was a turbid and eutrophic system with light availability controlled algal growth [15,60]. However, this ecological function was affected by the construction of upper weirs. Significant differences were found in TSI(CHL-a)-TSI(SD) and TSI(CHL-a)-TSI(TP) between post- and pre-construction periods (*p* < 0.05 and *p* < 0.001, respectively, Figure 7). The proportions of dissolved color clay particles and blue-green algae in TSID increased after the structures of upstream serial weirs, while the proportions of zooplankton grazing and non-algal turbidity decreased (Supplementary Table S4). Moreover, deviations in the TSI (CHL-a)-TSI (SD) observably decreased after weir constructions, indicating a decrease in inorganic contents in TSS in the system [28,31].

## 4. Conclusions

Consistent with the loss of reservoir water quality and the construction of serial upper weirs, we analyzed here the 19-year time series of seasonal water quality data in three longitudinal zones of the estuarine reservoir. First, our study showed that seasonal and spatial variations in water quality in the system are typically regulated by the hydroclimatic regime (intense precipitation and water turnover) and longitudinal zonation and that the variations were altered by the construction of serial upper weirs (which became operational in 2011). The deterioration of water quality in the reservoir was seasonally caused by monsoon rainfall runoff, the intensity of Asian monsoon, and sediment resuspension due to water turnover in autumn. These were also linked with intensive human activities within the catchment, which resulted in high levels of inorganic and organic matter, fecal coliform bacteria, and suspended solids during summer and autumn. Second, the statistical analyses showed that light availability was a key parameter for algal growth, which is directly controlled by non-algal turbidity. Winter algal blooms were observed in the estuarine reservoir due to the low level of inorganic turbidity and high phosphorus availability. Third, we found that the construction of serial weirs at upstream reaches could affect the nutrients, suspended solids, CHL-a level, and trophic state in the estuarine reservoir, resulting in lower TP and TN but high CHL-a and high TN:TP ratios. Moreover, the relationships between trophic state parameters in the TSID could be changed by the anthropogenic influence of serial upper weirs. However, the variations might also connect with the change in rainfall intensity during study years. Overall, we suggest that serial upstream disturbances such as weirs and dams are likely to cause the changes in the trophic state variables and their relationships in estuarine lentic systems, along with impacts of rainfall intensity and seasonal flow. Accordingly, the study can make an important contribution to appropriate water quality management in the estuarine reservoir. 

## Figures and Tables

**Table 1:** Loadings of water quality parameters in the first four principal components (PC 1, PC 2, PC 3, and PC 4).

Parameter	PC 1	PC 2	PC 3	PC 4
WT	−0.73	0.06	−0.44	0.50
EC	0.91	−0.32	−0.02	−0.17
TSS	−0.91	−0.08	−0.06	0.16
Kna	−0.93	−0.07	−0.08	−0.22
SD	0.95	−0.02	−0.14	0.22
TN	0.99	0.08	0.00	0.07
TDN	0.98	0.11	−0.02	0.10
NH_4_-N	0.92	0.20	0.01	0.09
NO_3_-N	0.98	0.01	−0.09	−0.05
TP	−0.17	0.64	0.72	−0.08
TDP	0.11	0.98	0.09	−0.13
PP	−0.46	−0.33	0.79	0.14
PO_4_-P	−0.23	0.87	0.11	−0.09
PP:TP	−0.43	−0.64	0.57	0.19
TN:TP	0.97	−0.10	−0.07	0.14
FCB	−0.81	0.25	−0.27	−0.29
CHL-a	0.30	0.31	0.66	0.40
PREC	−0.49	0.42	−0.37	0.65
**Eigenvalue**	**10.12**	**3.17**	**2.36**	**1.21**
**% variance**	**56.21**	**17.60**	**13.09**	**6.73**

**Table 2:** Changes in physicochemical parameters in the reservoir due to the construction of upper weirs.

Category	Parameter	Mean		ANOVA on Ranks	
		BWCs	AWCs	H Value	*p*-Value
Nutrient	TN (mg/L)	3.8	3.2	18.48	*
	TP (µg/L)	125	67	90.88	*
	TDP (µg/L)	96	38	92.22	*
	PP (µg/L)	28	28	0.04	
Ratios of Nutrients	TDP:TP	0.79	0.57	39.42	*
	PP:TP	0.21	0.43	39.42	*
	TN:TP	30.3	51.9	32.06	*
Algal biomass indicator	CHL-a (µg/L)	7.7	12.7	18.70	*
Ratios of CHL-a and Nutrients	CHL:TP	0.059	0.2	63.50	*
	CHL:TN	0.002	0.003	11.90	*
Suspended Solids/inorganic turbidity	TSS (mg/L)	12.65	16.9	7.08	*
	Kna (m^−1^)	1.48	1.41	1.01	

* Significance value (*p*) was lesser than 0.01, TN—total nitrogen, TP—total phosphorus, TDP—total dissolved phosphorus, PP—particulate phosphorus, CHL-a—chlorophyll-a, TSS—total suspended solids, Kna—non-algal turbidity.

## Data Availability

The data may be available upon request to the corresponding author, subject to approval.

## Supplementary Materials

The following are available online at https://www.mdpi.com/article/10.3390/ijerph182312568/s1, Figure S1: Biplot showing interrelation among water quality parameters in the estuarine reservoir (PP-particulate phosphorus, PREC-precipitation), Table S1: Spatial heterogeneities of water quality parameters during 2002–2020, along with range, average, and standard error (SE), Table S2: Spatial-seasonal variations of water quality parameters during 2020, along with ranges, averages, and standard error (SE), Table S3: Spatial and spatial-seasonal comparisons of water quality parameters in the reservoir during 2002–2020, Table S4: The variations in the TSID of the estuarine reservoir altered by the construction of new upper weirs.
