# Supplementary material for: Long-Term Water Quality Patterns in an Estuarine Reservoir and the Functional Changes in Relations of Trophic State Variables Depending on the Construction of Serial Weirs in Upstream Reaches
Source: Int J Environ Res Public Health. 2021 Nov 29;18(23):12568. doi: 10.3390/ijerph182312568 (PMC8656708; doi:10.3390/ijerph182312568)
Supplement: Supplementary file 1 [file ijerph-18-12568-s001.zip › ijerph-1420808-supplementary.pdf]

Supplementary Materials

Supplementary Table S1. Spatial heterogeneities of water quality parameters during 2002-2020, along with range, average, and standard error (SE).

Category	Parameter (unit)	Rz		Tz		Lz	
		**Mean±SE**	**(Range)**	**Mean±SE**	**(Range)**	**Mean±SE**	**(Range)**
Physical factors	WT(°C)	16.79±0.59	(1-33.9)	15.9±0.55	(2.2-30.5)	16.15±0.56	(1.7-30.2)
	EC (μS/cm)	558.81±38.59	(109-3422)	923.92±52.81	(100-4726)	1139.26±53.73	(100-5855)
	TSS (mg/L)	24.26±1.51	(1.6-223.6)	18.73±0.99	(1.9-138.7)	11.51±0.63	(1.2-91.4)
	SD (m)	0.52±0.02	(0.0-2)	0.65±0.03	(0.0-2.5)	1.02±0.05	(0.0-4.5)
	K_{na} (m^{-1})	1.19±0.11	(0.65-1.72)	1.35±0.15	(0.74-2.1)	0.91±0.13	(0.37-1.62)
Chemical factors	pH	7.84±0.04	(6.0-9.6)	7.63±0.03	(6.4-9.2)	7.74±0.03	(6.6-9.3)
	DO (mg/L)	10.02±0.21	(3.6-21.1)	8.78±0.2	(2.8-18.5)	9.61±0.19	(3.9-17.2)
	BOD (mg/L)	2.67±0.09	(0.3-8.2)	2.01±0.07	(0.2-7.9)	1.59±0.07	(0.3-8.8)
	COD (mg/L)	6.6±0.11	(2.9-12.5)	6.03±0.08	(3.1-9.4)	5.77±0.07	(2.6-9.9)
	TN (mg/L)	3.92±0.1	(0.86-10.17)	3.63±0.09	(1.2-9.26)	3.48±0.09	(1.09-8.35)
	TDN (mg/L)	3.52±0.09	(0.57-9.69)	3.25±0.08	(1.09-8.14)	3.11±0.08	(1.02-6.88)
	NH_4-N (mg/L)	0.42±0.03	(0.0-2.8)	0.32±0.02	(0.0-2.55)	0.25±0.02	(0.0-2.71)
	NO_3-N (mg/L)	2.32±0.07	(0.0-5.5)	2.25±0.06	(0.05-5.79)	2.2±0.06	(0.05-5.34)
	TP (μg/L)	129.91±5.43	(14-676)	107.91±4.33	(17-487)	92.04±5.14	(16-920)
	TDP (μg/L)	82.78±4.08	(7-542)	76.63±5.37	(7-264)	65.93±3.15	(3-291)
	PO_4-P (μg/L)	64.4±3.9	(0.0-515)	56.36±3.05	(0-249)	51.07±2.91	(0-259)
	TN:TP	39.1±1.72	(5.8-134.5)	45.1±2.01	(9.6-148.7)	55.9±2.82	(5.0-264.4)
Biological factors	FCB (col/100ml)	190.7±43.6	(0-4967)	186.7±48.9	(0-5861)	110.2±28.1	(0-3467)
	CHL-a (μg/L)	21.87±1.61	(0.0-173.4)	12.37±0.94	(0.01-99.2)	9.56±0.64	(0.1-60.8)

Rz – riverine zone, Tz – transition zone, Lz – lacustrine zone

Supplementary Table S2. Spatial-seasonal variations of water quality parameters during 2020, along with ranges, averages, and standard error (SE).

	Parameter (unit)	Rz				Tz				Lz			
		Spring	Summer	Fall	Winter	Spring	Summer	Fall	Winter	Spring	Summer	Fall	Winter
		Mean±SE (Range)				Mean±SE (Range)				Mean±SE (Range)			
Physical factors	WT (°C)	14.25±0.69 (4.3-23.8)	26.95±0.47 (14-33.9)	20.51±0.65 (12-29)	5.45±0.32 (1-12.9)	12.6±0.62 (4-20.7)	24.66±0.44 (16.8-30.5)	20.54±0.63 (13-29)	5.81±0.35 (2.2-12)	12.74±0.65 (4-20.5)	25.38±0.38 (19.1-30.2)	20.69±0.62 (11.4-29.3)	5.78±0.37 (1.7-11.9)
	EC (μS/cm)	798.5±93.8 (141-3422)	272±23.9 (109-1186)	311.9±30.4 (110-1209)	852.8±93.4 (236-3379)	1379±132.1 (190-4726)	613.4±83.6 (100-2513)	469±52.2 (109-1769)	1233.5±85.0 (255-2799)	1513.6±92.5 (220-3661)	706.12±74.6 (108-2740)	798.7±96.2 (100-5127)	1538±112.5 (449-5855)
	TSS (mg/L)	17.17±1.56 (2-62.8)	28.81±3.1 (8.8-161.6)	36.06±4.25 (2.4-223.6)	15.01±1.22 (1.6-63.6)	12.08±1.31 (3.7-70.9)	24.41±2.64 (8-138.7)	26.18±1.81 (6-64.3)	12.24±0.88 (1.9-35.3)	6.78±0.52 (2.2-28.6)	13.98±1.85 (2.5-91.4)	15.17±1.1 (2.4-52.4)	7.79±0.53 (1.2-20.4)
	SD (m)	0.68±0.06 (0.2-2)	0.44±0.03 (0.0-1.2)	0.35±0.03 (0.1-1.2)	0.6±0.04 (0.2-1.4)	0.87-0.07 (0.2-2.5)	0.52±0.04 (0.0-1.3)	0.47±0.04 (0.1-1.5)	0.74±0.04 (0.2-1.5)	1.57±0.13 (0.3-4.5)	0.87±0.1 (0.1-4)	0.63±0.04 (0.1-1.3)	1.01±0.05 (0.2-2)
	Kna (m^{-1})	0.87±0.07 (0.75-0.99)	1.28±0.2 (0.96-1.66)	1.61±0.07 (1.48-1.72)	1.01±0.23 (0.65-1.43))	0.89±0.04 (0.82-0.96)	1.67±0.26 (1.2-2.1)	1.85±0.08 (1.74-2.01)	0.99±0.19 (0.74-1.35)	0.41±0.02 (0.37-0.44)	1.17±0.2 (0.6-1.62)	1.42±0.05 (1.36-1.52)	0.77±0.15 (0.62-1.07)
Chemical factors	pH	7.89±0.06 (6.8-9.2)	8.03±0.1 (6.0-9.6)	7.63±0.07 (6.5-9.2)	7.83±0.08 (6.1-9.4)	7.78±0.05 (6.8-8.7)	7.59±0.07 (6.6-9.1)	7.43±0.05 (6.4-8.7)	7.72±0.07 (6.4-9.2)	7.94±0.05 (7.3-9.3)	7.74±0.08 (6.7-9.2)	7.52±0.04 (6.8-8.5)	7.75±0.06 (6.6-8.9)
	DO (mg/L)	10.69±0.28 (6.1-14.9)	7.21±0.25 (3.9-12.7)	7.98±0.21 (4.9-12.4)	12.57±0.26 (7.8-17.2)	9.92±0.3 (5-14.6)	6.08±0.24 (2.8-11.3)	7.01±0.19 (4.5-11.3)	12.11±0.29 (6.8-18.5)	10.42±0.32 (4.1-17.8)	8.24±0.33 (3.6-14.7)	7.98±0.22 (5.1-12.2)	13.42±0.38 (5.9-21.1)
	BOD (mg/L)	2.68±0.18 (0.5-6.1)	3.27±0.22 (0.9-8.2)	2.16±0.11 (0.9-4)	2.55±0.15 (0.3-5)	2.18±0.16 (0.2-6.3)	2.19±0.15 (0.7-7.9)	1.78±0.11 (0.5-4.2)	1.9±0.12 (0.5-4.6)	2.11±0.21 (0.3-8.8)	1.65±0.13 (0.3-6.8)	1.1±0.07 (0.3-2.5)	1.52±0.11 (0.3-3.6)
	COD (mg/L)	6.59±0.18 (4.3-10.3)	7.38±0.24 (3.4-12.5)	6.08±0.19 (2.9-9.1)	6.37±0.22 (3-10.6)	6.03±0.12 (4.2-8.2)	6.58±0.18 (3.1-9.4)	5.72±0.16 (3.1-8.8)	5.79±0.15 (3.5-9.3)	5.82±0.12 (2.7-7.8)	6.41±0.17 (2.6-9.9)	5.4±0.14 (2.7-7.8)	5.46±0.12 (3.8-8.3)
	TN (mg/L)	4.82±0.21 (1.27-10.17)	3.21±0.14 (0.86-5.54)	2.84±0.11 (1.67-5.95)	4.79±0.19 (2.82-9.45)	4.61±0.17 (1.2-8.63)	3.26±0.14 (1.36-6.46)	2.56±0.1 (1.43-5.41)	4.08±0.19 (2.21-9.26)	4.54±0.17 (1.09-8.35)	3.25±0.15 (1.4-7.05)	2.42±0.11 (1.24-5.63)	3.73±0.17 (1.74-6.79)
	TDN (mg/L)	4.48±0.19 (1.22-9.69)	2.86±0.13 (0.57-5.04)	2.42±0.07 (1.36-3.83)	4.33±0.17 (2.49-8.6)	4.29±0.15 (1.09-8.06)	2.9±0.11 (1.16-5.44)	2.19±0.06 (1.27-3.22)	3.6±0.16 (2.11-8.14)	4.2±0.13 (1.02-6.88)	2.89±0.11 (1.12-5.71)	2.09±0.06 (1.22-3.69)	3.28±0.13 (1.61-6.41)
	NH_4-N (mg/L)	0.74±0.09 (0.01-2.8)	0.22±0.03 (0.00-1.04)	0.17±0.02 (0.00-0.7)	0.55±0.07 (0.00-2.49)	0.58±0.06 (0.03-2.55)	0.18±0.03 (0.01-0.91)	0.15±0.03 (0.00-1.16	0.36±0.04 (0.00-1.82)	0.49±0.06 (0.03-2.71)	0.14±0.03 (0.00-1.17)	0.1±0.02 (0.00-0.83)	0.28±0.04 (0.00-1.52)
	NO_3-N (mg/L)	2.98±0.12 (1.52-5.5)	1.66±0.11 (0.00-4.33)	1.78±0.08 (0.1-3.32)	2.85±0.11 (0.03-5.39)	2.99±0.12 (0.81-5.79)	1.94±0.11 (0.07-3.95)	1.56±0.06 (0.1-2.76)	2.49±0.11 (0.05-5.4)	3.02±0.1 (1.12-5.34)	1.95±0.1 (0.05-3.6)	1.54±0.06 (0.08-2.71)	2.29±0.11 (0.1 - 4.78)
	TP (μg/L)	124.4±10 (14-380)	127.6±6.76 (25-228)	126.7±5.92 (54-250)	140.9±17.16 (32-676)	94.5±7.68 (17-264)	118.1±8.79 (28-428)	113.7±6.1 (43-250)	105.4±11.17 (27-487)	81.2±8.36 (16-303)	93.9±6.79 (20-194)	97.5±6.46 (31-271)	95.5±16.37 (23-920)

	TDP (µg/L)	81.4±7.9 (11-210)	85.6±6.51 (7-195)	74.7±4.95 (21-172)	89.5±4.14 (8-542)	83.6±18.31 (12-240)	80.4±5.91 (7-213)	69.7±4.91 (21-154)	72.8±8.37 (12-264)	66.6±8.14 (10-291)	68.6±5.88 (3-189)	67.8±4.14 (15-148)	60.8±6.51 (12-217)
	PO_4-P (µg/L)	63.7±7.75 (1-200)	65.7±6.24 (0.00-185)	60.5±4.83 (0.00-159)	67.8±11.11 (0.00-515)	52.9±6.48 (0.00-192)	62.5±5.3 (3-210)	55.2±4.52 (4-126)	54.9±7.74 (0.00-249)	48.6±7.22 (0.00-259)	53.3±5.48 (1-184)	54.8±4.03 (0.00-132)	47.6±6.21 (0.00-175)
	TN:TP	49.2±3.44 (17.1-134.5)	30.8±2.58 (5.8-108.3)	24.7±1.26 (10.5-53.4)	51.9±4.14 (7.8-134.2)	64.7±4.53 (23.-148.7)	35.5±2.89 (9.9-114.2)	25.45±1.47 (10.9-74.7	54.8±4.16 (9.6-146.2)	84.2±7.12 (16.5-264.4)	50.7±5.22 (10.8-189.4)	28.1±1.35 (8.9-55.3)	60.4±4.47 (5.0-149.5)
Biological factors	FCB (col/100ml)	98.1±48.61 (0-2283)	238±98.39 (0-4000)	270.8±94.02 (0-3467)	155.2±98.7 (0-4967)	69±43.31 (0-2395)	263.5±126.9 (0-5861	284.5±108.1 (0-3800)	128.7±91.8 (0-5069)	39.8±24.9 (0-1376)	140.6±61.4 (0-1947)	167.9±65.2 (0-2400)	92.3±63.3 (0-3467)
	CHL-a (µg/L)	18.7±3 (1.6-112.8)	26.9±3.31 (0.7-109.8)	15.56±1.59 (0.2-53.7)	26.3±4.24 (0.0-173.4)	10.29±1.27 (0.7-47.4)	12.85±1.69 (0.3-60.2)	11.05±1.26 (0.01-57.3)	15.29±2.82 (0.1-99.2)	9.28±1.23 (0.5-40.7)	11.37±1.64 (0.2-60.8)	8.08±0.89 (0.1-29.3)	9.49±1.21 (0.2-44.7)

Rz – riverine zone, Tz – transition zone, Lz – lacustrine zone.

Supplementary Table S3. Spatial and spatial-seasonal comparisons of water quality parameters in the reservoir during 2002 – 2020.

		Parameter (unit)	WT	EC	TSS	SD	K_{na}	pH	DO	BOD	COD	TN	TDN	NH_4-N	NO_3-N	TP	TDP	PO_4-P	TN:TP	FCB	CHL-a
**Spatial Comparison**	ANOVA on Ranks	***H value***	1.23	94.09	113.05	100.2	81.82	20.97	17.57	112.66	37.05	11.49	10.18	19.02	1.37	48.45	10.06	5.32	20.94	10.87	58.06
		p-value	>0.05	**<0.001**	**<0.001**	**<0.001**	**<0.001**	**<0.001**	**<0.001**	**<0.001**	**<0.001**	**<0.01**	**<0.01**	**<0.001**	>0.05	**<0.001**	**<0.01**	>0.05	**<0.001**	**<0.01**	**<0.001**
	t-test (*p<0.05**)	Rz vs Tz		*	*	*	*		*	*	*					*					*
		Rz vs Lz		*	*	*	*	*		*	*	*	*	*		*	*		*	*	*
		Tz vs Lz		*	*	*	*	*	*	*	*			*		*			*		*
**Seasonal Comparison on each site** **Lz**	ANOVA on Ranks	***H value***	176.08	76.53	73.35	60.88	44.78	32.89	134.26	22.49	29.61	90.46	114.24	61.13	96.85	9.78	6.59	6.85	67.54	11.23	1.33
		p-value	**<0.001**	**<0.001**	**<0.001**	**<0.001**	**<0.001**	**<0.001**	**<0.001**	**<0.001**	**<0.001**	**<0.001**	**<0.001**	**<0.001**	**<0.001**	**<0.05**	>0.05	>0.05	**<0.001**	**<0.05**	>0.05
	t-test (*p<0.05**)	S vs Su	*	*	*	*	*	*	*			*	*	*	*				*		
		Su vs F	*							*	*	*	*		*				*		
		F vs W	*	*	*	*	*	*	*	*		*	*	*	*				*		
		W vs S	*			*	*		*			*	*	*	*						
		S vs F	*	*	*	*	*	*	*	*		*	*	*	*	*			*	*	
		W vs Su	*	*	*				*		*			*							
**Tz**	ANOVA on Ranks	***H value***	173.36	73.98	77.65	40.12	21.12	27.58	138.56	5.75	25.93	96.7	114.96	58.47	90.81	10.66	6.14	6.48	75.14	17.21	1.51
		p-value	**<0.001**	**<0.001**	**<0.001**	**<0.001**	**<0.001**	**<0.001**	**<0.001**	>0.05	**<0.001**	**<0.001**	**<0.001**	**<0.001**	**<0.001**	**<0.05**	>0.05	>0.05	**<0.001**	**<0.001**	>0.05
	t-test (*p<0.05**)	S vs Su	*	*	*	*	*	*	*			*	*	*	*				*		
		Su vs F	*								*	*	*		*						
		F vs W	*	*	*	*	*	*	*			*	*	*	*				*	*	
		W vs S	*						*				*								
		S vs F	*	*	*	*	*	*	*			*	*	*	*				*	*	
		W vs Su	*	*	*	*			*		*	*	*	*	*				*		
**Rz**	ANOVA on Ranks	***H value***	178.26	94.03	53.31	40.73	22.94	15.39	103.32	14.36	53.02	98.12	110.64	56.57	93.72	2.39	1.39	1.39	54.71	20.03	6.16
		p-value	**<0.001**	**<0.001**	**<0.001**	**<0.001**	**<0.001**	**<0.01**	**<0.001**	**<0.01**	**<0.001**	**<0.001**	**<0.001**	**<0.001**	**<0.001**	>0.05	>0.05	>0.05	**<0.001**	**<0.001**	>0.05
	t-test (*p<0.05**)	S vs Su	*	*	*	*			*		*	*	*	*	*				*		
		Su vs F	*					*		*	*										
		F vs W	*	*	*	*	*		*			*	*	*	*				*	*	
		W vs S	*						*												
		S vs F	*	*	*	*	*	*	*			*	*	*	*				*	*	
		W vs Su	*	*	*	*			*		*	*	*	*	*				*		

Lz-lacustrine zone, Tz–transition zone, Rz–riverine zone; S–spring, Su-summer, F-fall, W-winter; *p<0.05.

Supplementary Table S4. The variations in the TSID of the estuarine reservoir altered by the construction of new upper weirs.

	TSID plot statement	Whole year (%)	BWCs(%)	AWCs (%)
P-I	Smaller particles predominate (P - limitation)	4.73	0.31	8.71
P-II	Larger particles predominate (Blue green algae)	5.62	1.56	9.27
P-III	Non-algal light limitation	82.99	87.81	78.65
P-IV	Zooplankton grazing	6.66	10.31	3.37

BWCs-before upper weir constructions, AWCs –after upper weir constructions

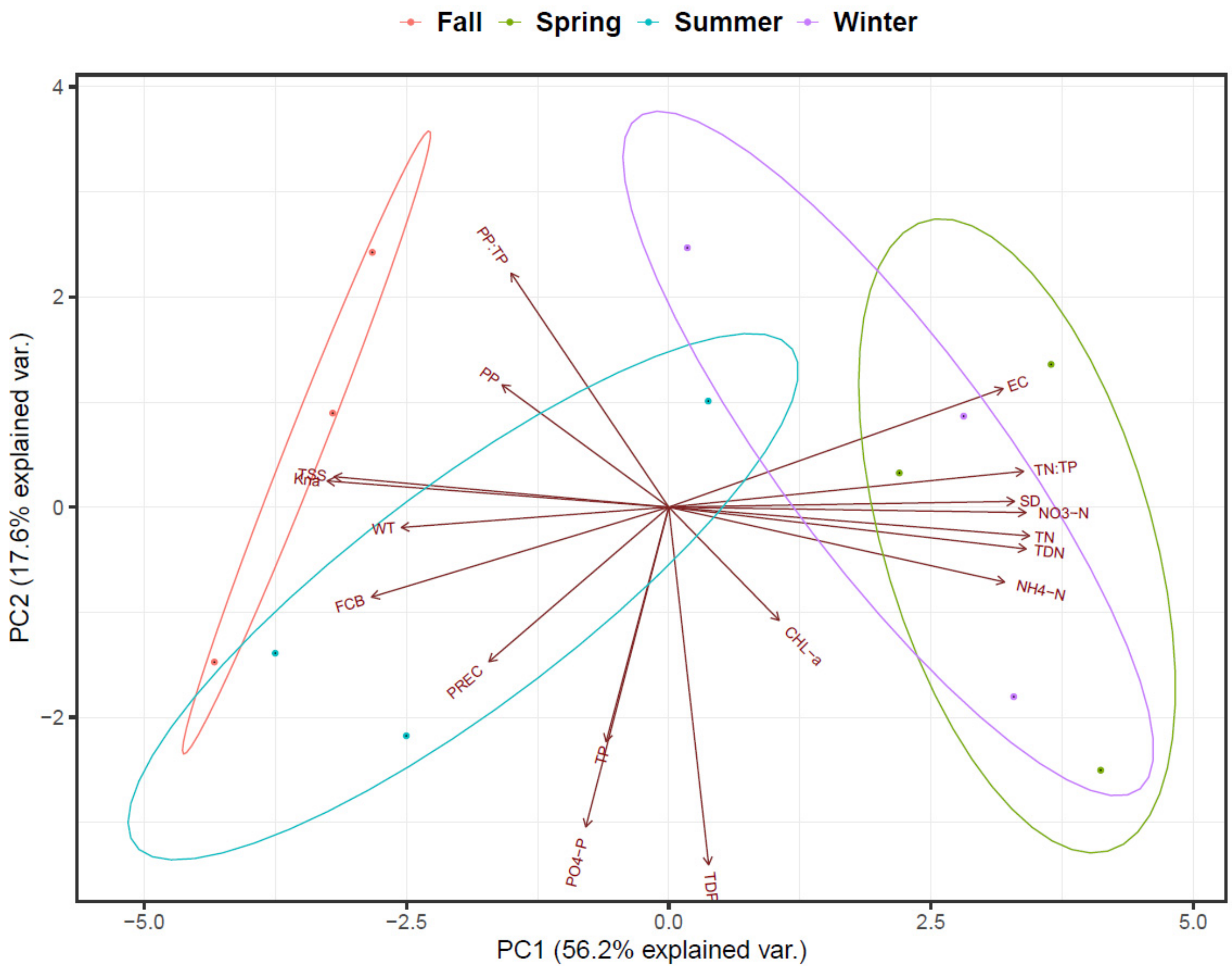

Supplementary Figure S1. Biplot showing interrelation among water quality parameters in the estuarine reservoir (PP-particulate phosphorus, PREC-precipitation).
